# Global research trends and hotspots evolution in type 2 diabetes mellitus complicated with coronary heart disease: a bibliometric analysis from 2016 to 2026

**DOI:** 10.3389/fendo.2026.1900634

**Published:** 2026-08-04

**Authors:** Chunying He, Xiaohua Hu, Xiaolan Yin, Min Zhan, WeiYi Cao, Rui Li

**Affiliations:** 1 Institute of Clinical Pharmacology of Xiyuan Hospital, National Clinical Research Center for Chinese Medicine Cardiology, China Academy of Chinese Medical Sciences, Beijing, China; 2 NMPA Key Laboratory for Clinical Research and Evaluation of Traditional Chinese Medicine, National Clinical Research Center for Chinese Medicine Cardiology, Beijing, China; 3 Department of Digestive Diseases, Xiamen Traditional Chinese Medicine Hospital, Xiamen, China; 4 Department of Neurology, China Academy of Chinese Medical Sciences Xiyuan Hospital, Beijing, China

**Keywords:** bibliometrics, coronary heart disease, development trends, research hotspots, type 2 diabetes mellitus

## Abstract

**Background:**

The comorbidity burden of type 2 diabetes mellitus complicated with coronary heart disease is steadily increasing. However, there is a lack of systematic mapping of the research landscape, knowledge foundation, and emerging frontiers in this field.

**Methods:**

A systematic search of the Web of Science Core Collection, PubMed, and Scopus was conducted for literature published from April 2016 to April 2026. A series of tools, such as Origin Pro 2025, SCImago Graphica 1.0.55, VOSviewer 1.6.20, Bibliometrix R 4.6.0, and SciExplorer, were used to analyze annual trends, countries/regions, institutions, authors, journals, highly cited references, keyword co-occurrence, and thematic evolution.

**Results:**

A total of 8,390 publications were included, with annual output showing sustained growth. The United States and China emerged as the dominant contributors, and the United States displayed markedly higher international collaboration rates and greater citation impact per article. Institutions such as Harvard University and the University of Toronto anchored the core collaborative network. *Cardiovascular Diabetology* was identified as the most prolific journal. Highly cited documents predominantly comprised clinical guidelines and comprehensive reviews. Polygenic risk scores, machine learning applications, and the cardiovascular advantages of novel glucose-lowering agents are current research hotspots. Multi-omics investigations and integrated cardiometabolic multimorbidity management are gaining recognition as promising frontier directions.

**Conclusion:**

The past decade has witnessed a surge of research progress and innovation in this field. Capitalizing on the clinical translation of metabolomics, artificial intelligence-assisted decision-making, and integrated multimorbidity management represents a feasible pathway toward breakthrough research.

## 1 Introduction

Patients with concomitant type 2 diabetes mellitus (T2DM) and coronary heart disease (CHD) are consistently recognized as being at exceptionally high risk for recurrent cardiovascular events and mortality (1, 2). Diabetes constitutes a major risk factor for both the occurrence of a first cardiovascular event and subsequent worsening of prognosis (3, 4). Current evidence indicates that the presence of diabetes substantially alters clinical outcomes in patients with cardiovascular disease, conferring an overall 40% increase in major adverse cardiovascular events compared with individuals without diabetes (5).

Over the past decade, the management of T2DM co-occurring with CHD has attracted intensifying scholarly attention, generating a substantial body of research that has considerably advanced the field (6, 7). In terms of risk stratification, researchers have pursued more refined definitions and stratification of high-risk populations, thereby fostering the development of early risk management strategies (8). Regarding pathological mechanisms, atherosclerotic plaques in patients with diabetes have been demonstrated to be more prone to rupture than those in non-diabetic individuals, placing them at elevated cardiovascular event risk (9). In the therapeutic domain, intensive antithrombotic therapy has shown greater clinical benefit in patients with diabetes than in those without, while the mode of glycemic management itself exerts a profound influence on cardiovascular outcomes (10–13).

Great progress has been made in many fields in the last decade. But the existing research is mostly fragmented in individual subtopics, and there is a lack of systematic mapping of the overall knowledge landscape and macroscopic understanding of frontier directions (14, 15).With hundreds of publications appearing annually, navigating the literature, identifying core themes, and pinpointing the most promising directions have become genuinely demanding tasks.

Bibliometrics, as a quantitative approach to delineating the developmental dynamics of a discipline, offers an effective tool for overcoming these challenges (16). Through the quantitative analysis and synthesis of data, it can construct a coherent knowledge framework, revealing the spatiotemporal distribution of knowledge production and the evolutionary trajectories of research frontiers. Bibliometrics offers three distinct advantages over conventional review approaches. First, it enables large-scale literature processing. It can efficiently handle datasets comprising thousands to tens of thousands of publications, exceeding the volume capacity of manual reviews. This allows comprehensive coverage of a research field and reduces conclusion bias caused by limited sample selection. Second, it ensures objective and reproducible findings. All analyses are built on verifiable bibliometric indicators and standardized algorithms. Quantitative data are used to reflect research output, academic impact, and thematic distribution, which minimizes bias from subjective interpretation and makes the entire research workflow reproducible. Third, it supports visual mapping of knowledge structures. Using techniques such as co-occurrence analysis and network construction, it intuitively demonstrates academic collaboration networks, knowledge clusters, and the evolution of research hotspots. It can also uncover emerging frontiers and interdisciplinary links that are difficult to identify through traditional qualitative reviews. Therefore, we performed a systematic bibliometric analysis using integrated records from three major databases: Web of Science Core Collection (WOSCC), PubMed, and Scopus. This study aims to comprehensively characterize the global research landscape, knowledge base, and evolving frontiers of T2DM co-occurring with CHD, and to inform future research and clinical practice in this field.

## 2 Materials and methods

### 2.1 Data sources and search strategy

A systematic literature search was performed across three major electronic databases—the Web of Science Core Collection (WoSCC), Scopus, and PubMed—to identify studies examining CHD and T2DM. The search was limited to publications published from April 1, 2016, to April 1, 2026.

The search strategy adopted standardized MeSH and keywords related to CHD and T2DM (details provided in **Table 1**). Distinct retrieval fields were employed in accordance with the rules of each database: Topic (TS) for WoSCC, TITLE - ABS - KEY for Scopus, and Title/Abstract for PubMed.

**Table 1:** Search queries of T2DM complicated with CHD.

Rank	Search term
#1	“Diabetes Mellitus, Type 2” OR “Diabetes Mellitus, Noninsulin-Dependent” OR “Stable Diabetes Mellitus” OR “Diabetes Mellitus, Type II” OR “NIDDM” OR “Diabetes Mellitus, Noninsulin Dependent” OR “Type 2 Diabetes Mellitus” OR “Noninsulin Dependent Diabetes Mellitus” OR “Type 2 Diabetes” OR “Diabetes, Type 2” OR “Diabetes Mellitus, Non-Insulin Dependen” OR “Diabetes Mellitus, Maturity-Onset” OR “Diabetes Mellitus, Maturity Onset” OR “Maturity-Onset Diabetes Mellitus” OR “Maturity Onset Diabetes Mellitus” OR “MODY” OR “Diabetes Mellitus, Slow-Onset” OR “Diabetes Mellitus, Slow Onset” OR “Diabetes Mellitus, Adult-Onset”
#2	“Coronary Disease” OR “Coronary Artery Disease” OR “Disease Coronary” OR “Coronary Heart Disease” OR “Disease, Coronary Heart” OR “Heart Disease, Coronary”
#3	#1 AND #2
#4	Document types other than articles and reviews were excluded; Records outside the medical and related subject areas were removed; Publications not written in English were also excluded.

### 2.2 Data processing and analysis

**Figure 1:**
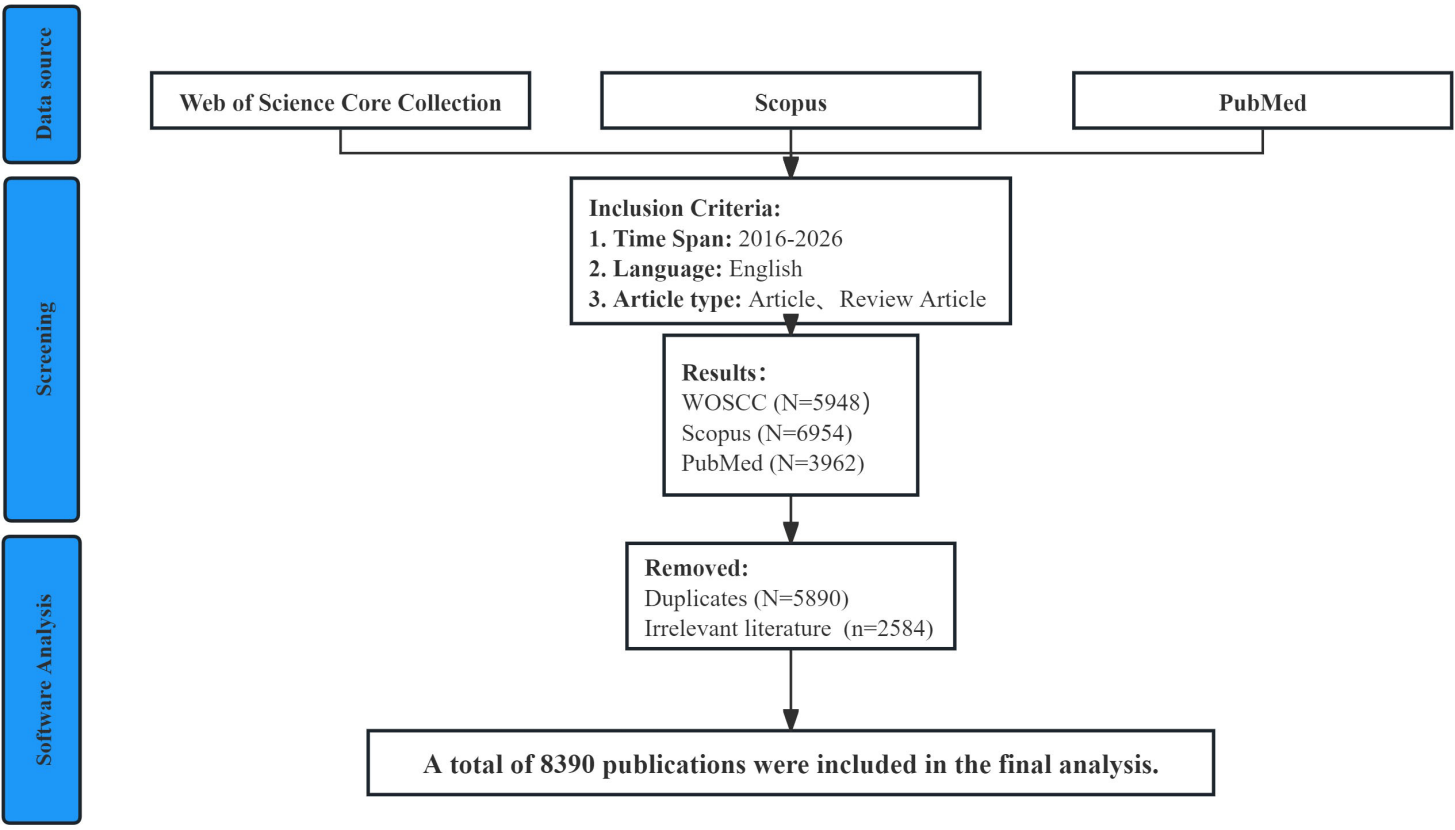
Flowchart of literature screening.

Records retrieved from WoSCC were exported as plain text files; those from Scopus were exported as both CSV and RIS files; and records from PubMed were exported in the native PubMed format. Records exported from the three databases were imported into NoteExpress software. Deduplication was performed using DOI as the primary matching criterion for precise matching. For records without a DOI, a combination of “title + first author surname + publication year” was used for fuzzy matching to initially remove duplicates. Manual verification was then conducted: two researchers independently cross-checked the deduplicated results, with special attention to records with similar titles or incomplete information. After confirming no omissions or errors, the final literature set was formed, and further exclusion of records unrelated to the research topic was performed. The resulting literature set was merged across databases using the Bibliometrix R package (version 4.6.0). The field completeness after merging the three databases was verified. The results showed that, apart from citation information, other data loss was within an acceptable range. Since the Web of Science Core Collection (WOSCC) has the most uniform citation indexing rules and the highest data completeness, to ensure comparability and accuracy of citation indicators, all citation-related analyses in this study were based solely on WOSCC as the data source. Scopus and PubMed were only used for bibliographic analyses such as annual publication counts, country/institution distribution, and keyword co-occurrence, and were not included in any citation-related metric calculations. Ultimately, a total of 8,390 publications were included in the subsequent bibliometric analysis and saved in Rdata format. The detailed screening and selection workflow is presented in **Figure 1**.

### 2.3 Visualization and statistical tools

Annual publication trends were analyzed using Origin Pro 2025. The inter-country/regional collaboration network was depicted with SCImago Graphica 1.0.55. VOSviewer 1.6.20 was applied to perform visual analyses of institutions, journals, highly cited references, and keywords. The Bibliometrix R package (version 4.6.0) was employed to construct author collaboration networks, identify core journals, generate keyword word clouds, and carry out thematic evolution mapping. The online platform SciExplorer (https://smartdata.las.ac.cn/SciExplorer) was used to produce a “Categories overlay” plot, illustrating the distribution and evolution of research disciplines.

## 3 Results

### 3.1 Trend analysis of annual publication

**Figure 2:**
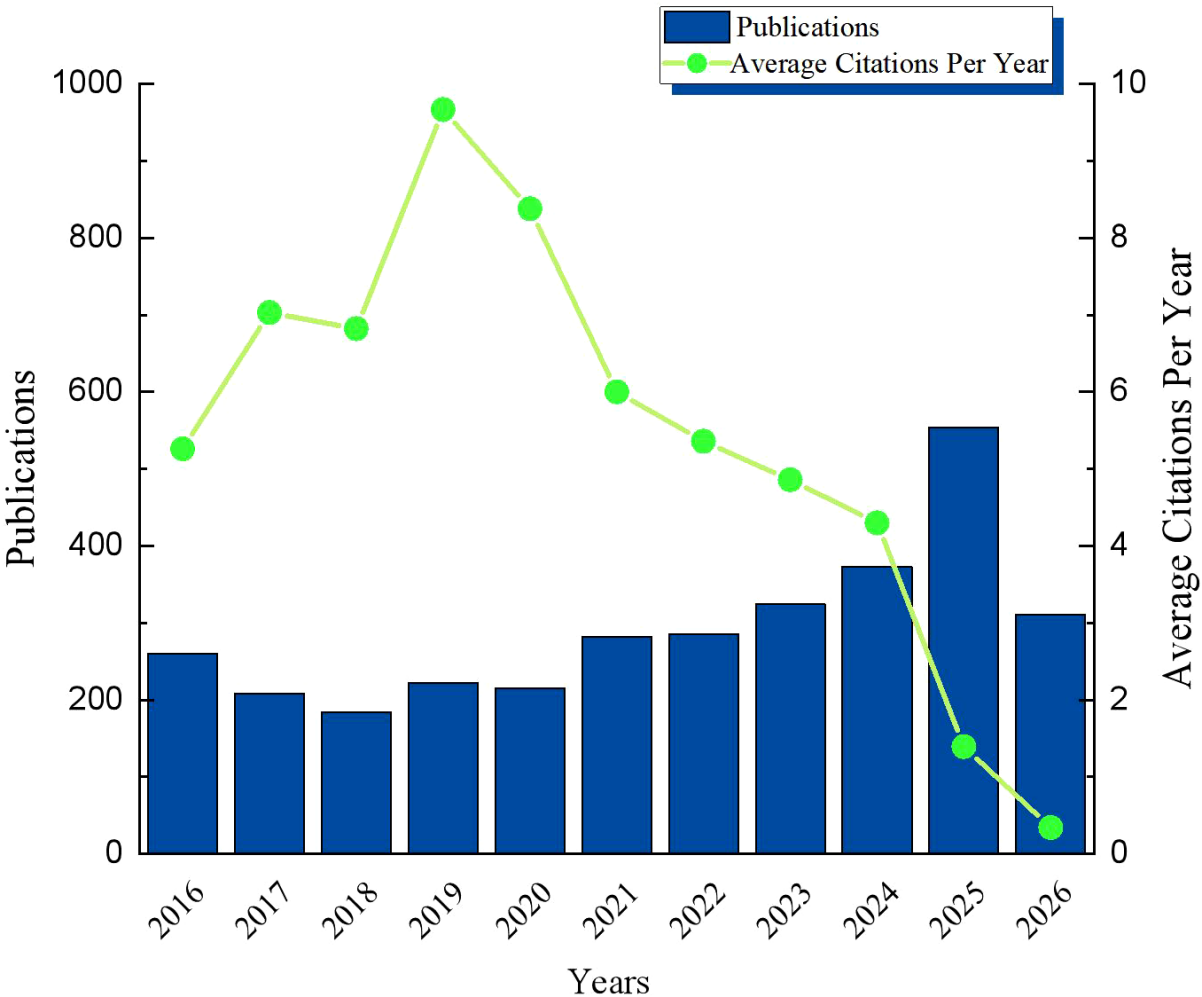
Annual publication growth and citations trend chart.

The annual number of publications grew steadily between 2016 and 2021, signaling sustained and increasing attention to this field. A sharp acceleration in output was observed from 2021 to 2025, reflecting the field’s emergence as a research hotspot within the cardiometabolic discipline. With respect to annual average citations, a continuous rise was recorded from 2016, peaking in 2019, which indicates that articles published during this window achieved particularly high scholarly impact. From 2020 to 2026, the average citation rate gradually declined. These trends are depicted in **Figure 2**.

### 3.2 Distribution and cooperation by country/region

**Figure 3:**
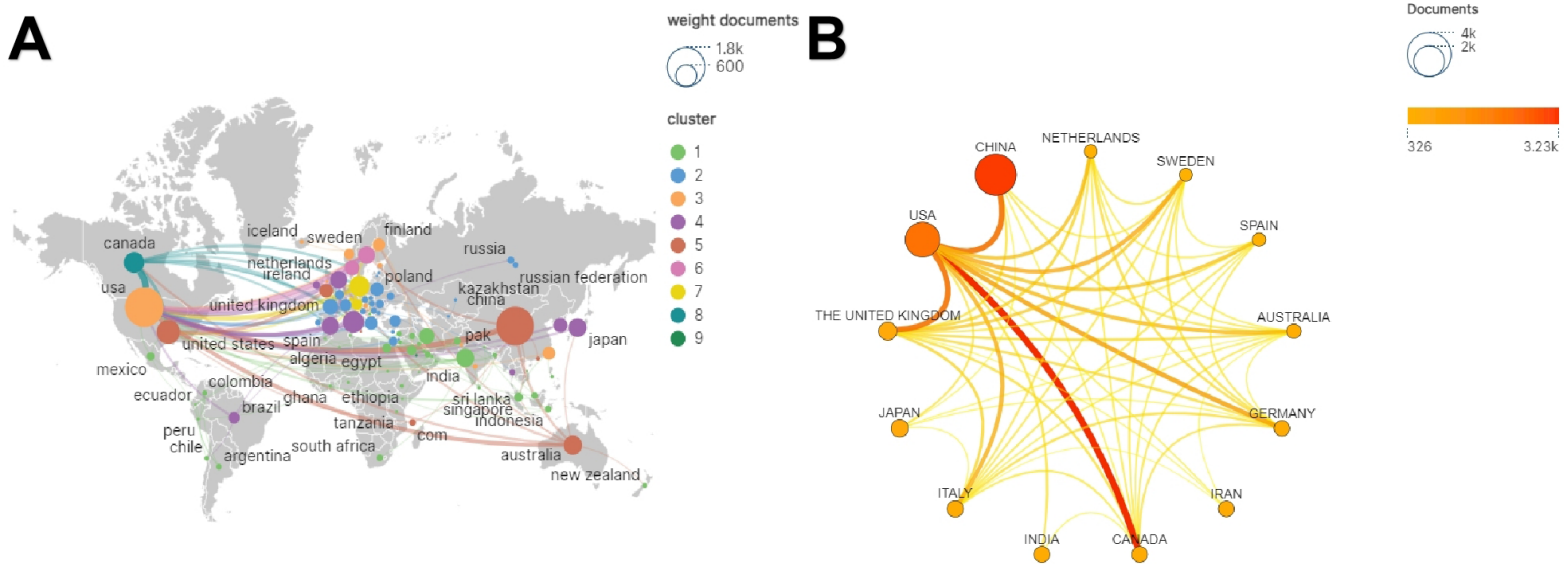
Publications and strength of partnership of the countries/regions. **(A)** Worldwide distribution of publications. **(B)** Partnership chord diagram.

In terms of research output, China and the United States ranked markedly ahead of all other countries. China contributed 1,563 articles (18.6%), while the United States contributed 1,330 articles (15.9%), together accounting for 34.5% of the global literature in this field. This dominant position indicates the high research investment and growing academic influence of both countries at the interface of cardiovascular and metabolic sciences. However, Chinese researchers are outperformed by their counterparts from the United States in terms of international collaborative activities and per-publication academic impact (**Figure 3A**).

Regarding international collaboration patterns, the United Kingdom demonstrated the highest level of multinational engagement, with a multiple-country publication (MCP) ratio of 44.0%. Other Western countries such as Canada also showed considerable collaboration with an MCP rate of 37.6%. These results suggest that native English-speaking countries are central hubs in the international research network, a pattern that can be explained by linguistic advantages, well-established transnational research platforms, and funding mechanisms that explicitly promote global partnerships (**Figure 3B**).

In terms of scholarly impact, the United Kingdom was the most impactful country, with an average of 78.5 citations per paper, which was significantly higher than the other countries. This outstanding performance is closely linked to its broad international collaboration (MCP 44.0%) and a deep-rooted reputation for excellence built up over decades of sustained research in this field (**Table 2**).

**Table 2:** The top 10 productive countries/regions.

Ranking	Country	Articles	Articles percentage %	SCP	MCP	MCP %	Average article citations
1	CHINA	1563	18.6	1392	171	10.9	18.6
2	USA	1330	15.9	982	348	26.2	66.7
3	THE UNITED KINGDOM	384	4.6	215	169	44	78.5
4	JAPAN	308	3.7	291	17	5.5	29.1
5	ITALY	302	3.6	241	61	20.2	43.2
6	INDIA	248	3	228	20	8.1	14.6
7	CANADA15	245	2.9	153	92	37.6	53.2
8	IRAN	239	2.8	188	51	21.3	20.6
9	GERMANY	230	2.7	154	76	33	41.5
10	SPAIN	201	2.4	141	60	29.9	44.6

SCP, Single country publication, i.e. the authors of the publication are from the same country. MCP, Multiple-country publications: The authors of the publication are from more than one country, which indicates the collaboration between countries. MCP %: the proportion of co-authored articles in the country’s publications.

### 3.3 The most productive institutions

As shown in **Table 3**, Harvard University ranked first with 311 publications, far exceeding the second-ranked University of Toronto (117 publications), demonstrating an overwhelming advantage in research productivity. In terms of academic impact, Harvard University (11,741 citations) exhibited a markedly superior performance, followed by the University of Toronto (7,592 citations), Massachusetts General Hospital (2,867 citations), and Monash University (2,280 citations).

**Table 3:** The top 15 institutions by publication volume.

Ranking	Institutions	Country	Count	Citations
1	Harvard University	USA	311	11741
2	University of Toronto	Canada	117	7592
3	Karolinska Institutet	Sweden	101	55
4	University of Oxford	UK	90	1515
5	Massachusetts General Hospital	USA	85	2867
6	Imperial College London	UK	84	167
7	University of Copenhagen	Denmark	83	577
8	Shanghai Jiao Tong University	China	82	2526
9	Tehran University of Medical Sciences	Iran	80	606
10	University of Glasgow	UK	75	258
11	Capital Medical University	China	74	1185
12	University College London	UK	72	480
13	Instituto de Salud Carlos III	Spain	69	198
14	University of Cambridge	UK	67	1443
15	Monash University	Australia	66	2280

USA, the United States of America; UK, the United Kingdom.

**Figure 4:**
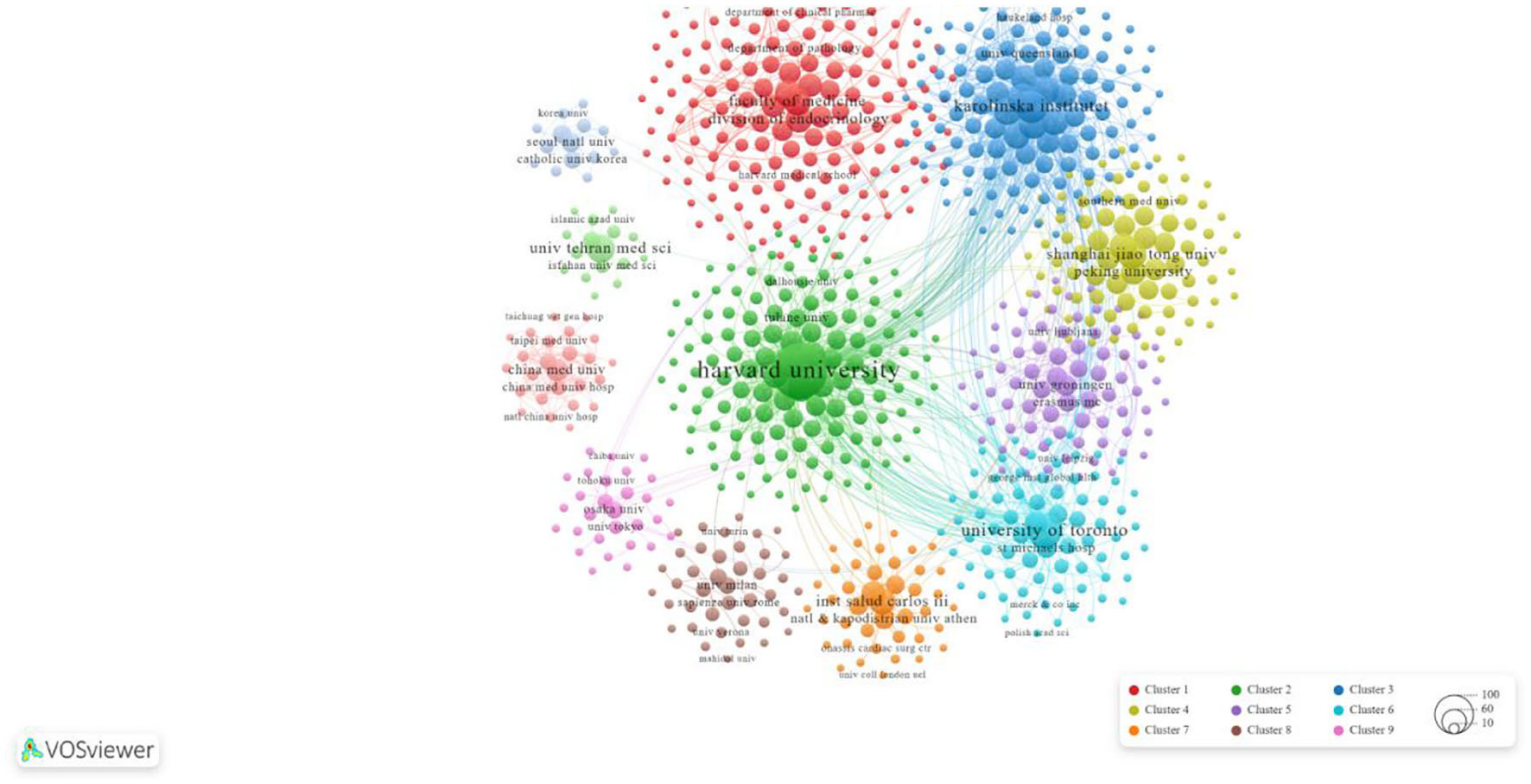
Institutional collaboration clustering network.

VOSviewer-based clustering of institutional collaboration networks further revealed the underlying structure of cooperative patterns in this field (**Figure 4**). The dense intra-cluster links reflect the formation of relatively stable cooperative communities among institutions, and the extensive inter-cluster connections suggest the active knowledge exchange among diverse research communities. From a network topology perspective, Harvard University is positioned at the geometric center of the entire network, exhibiting the largest node size and the most abundant inter-cluster connections. This indicates that, in addition to its substantial research output, Harvard serves as a pivotal “hub institution” within the international collaboration network, functioning as a critical bridge that connects diverse research communities and facilitates knowledge dissemination. In their respective clusters, the University of Toronto, the University of Oxford, and Shanghai Jiao Tong University serve as core nodes that drive the growth of regional cooperative subgroups. Notably, five UK institutions ranked among the top 15 (the University of Oxford, Imperial College London, the University of Glasgow, University College London, and the University of Cambridge) and were distributed across multiple clusters, reflecting the country’s broad institutional coverage, multi-center collaboration model, and a distinctive cluster-based research strength.

### 3.4 Contributions of authors

Based on multidimensional bibliometric indicators, this study systematically delineated the productivity landscape and impact differentiation among core authors in this field. As shown in **Table 4**, WANG Y ranked first with 163 publications, followed by LI Y (154) and ZHANG Y (147); these three authors constituted the leading echelon in terms of publication output. In terms of total citations (TC), LI Y exhibited a commanding lead with 7,119 citations, followed by WANG Y (4,109) and LI J (3,979).

**Table 4:** The top 10 productive authors.

Ranking	Author	Count	Total citations	h_index	g_index	m_index
1	WANG Y	163	4109	30	59	2.727
2	LI Y	154	7119	33	83	3
3	ZHANG Y	147	2990	25	50	2.273
4	LIU Y	122	2967	24	52	2.182
5	LI J	117	3979	27	62	2.455
6	CHEN Y	117	2449	24	45	2.182
7	WANG X	104	2140	21	44	1.909
8	WANG J	96	1217	19	30	1.727
9	LI X	93	2959	24	54	2.182
10	ZHANG J	89	1922	18	42	1.636

To avoid the well-known limitations of single-metric evaluation, we examined h-index, g-index, and m-index as complementary measures. The h-index balances publication count with citation quality by identifying the number of papers (h) that have each received at least h citations. The g-index extends this by capturing the cumulative citation depth of an author’s most influential works, calculated as the top g papers whose combined citations equal or exceed g². The m-index further adjusts the h-index for career duration, thereby estimating the annual rate at which scholarly influence accumulates.

With regard to the h-index, LI Y ranked first (h = 33), followed by WANG Y (h = 30) and LI J (h = 27), indicating that each of these authors possessed a substantial portfolio of highly cited core papers. However, the g-index uncovered meaningful distinctions: LI Y’s value of 83 substantially exceeded the corresponding h-index, suggesting a pronounced “peak effect” driven by a collection of highly cited landmark papers. By contrast, the narrower gap between h-index and g-index for other authors implied a more uniform, less concentrated citation distribution. LI Y also led in m-index (3.000), ahead of WANG Y (2.727) and LI J (2.455). The M-index metric effectively accounts for biases arising from variations in academic career length, indicating that LI Y has attained the highest annualized accumulation of impact within a relatively shorter duration, signifying robust academic momentum.

**Figure 5:**
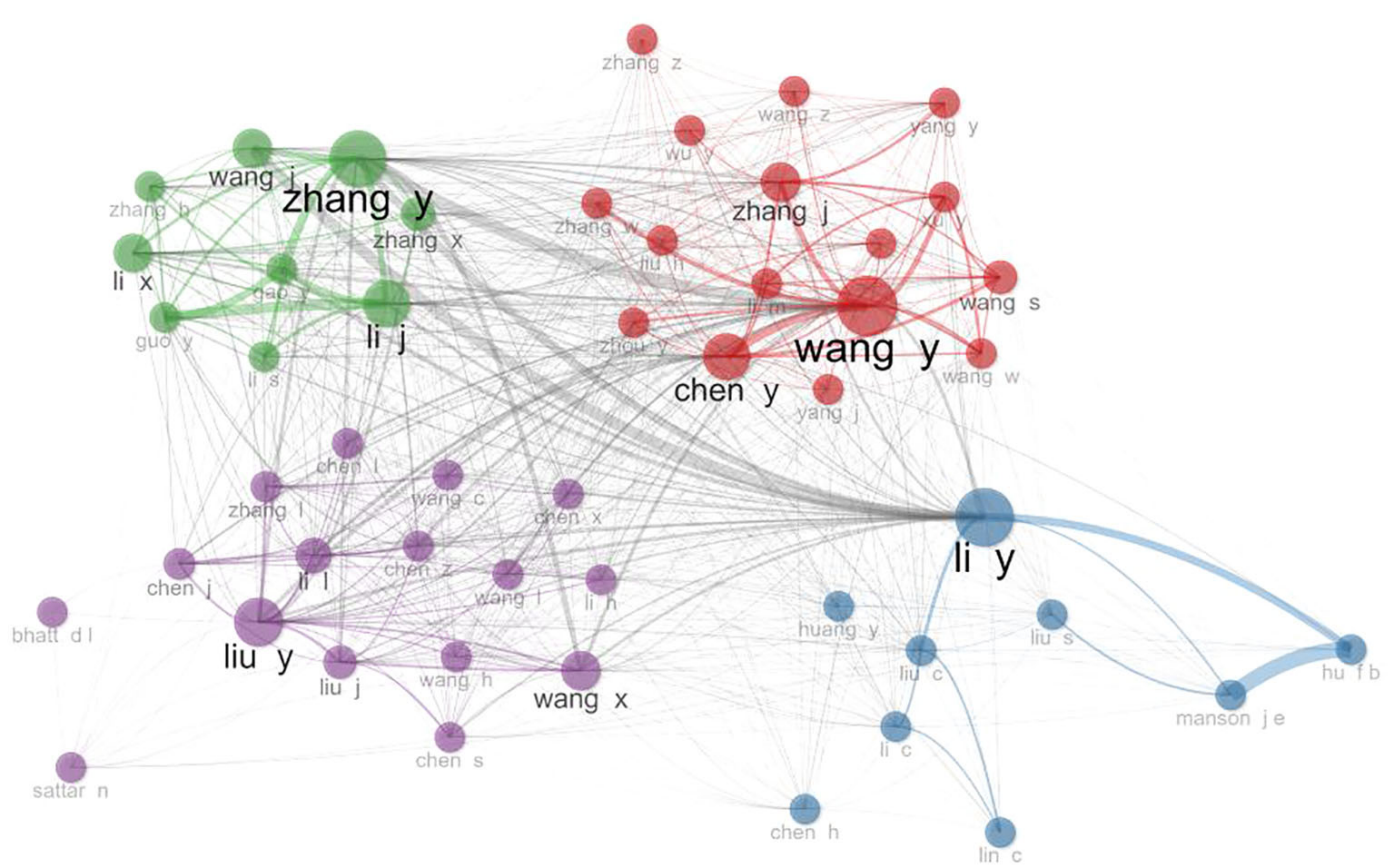
Core author collaboration network.

As illustrated in **Figure 5**, the red cluster was the largest and most densely packed, with WANG Y as the core node, constituting the most active collaborative subgroup in the field. The blue cluster, centered around LI Y and colleagues, was relatively smaller in size but exhibited tight internal connectivity. Notably, although LI Y led comprehensively in impact indicators, the cross-cluster links of his group within the network were not prominent, suggesting that his collaboration pattern may have revolved around intensive cooperation within a core team rather than extensive cross-group engagement. In contrast, the cluster containing WANG Y featured numerous nodes and dense connections, indicating that high productivity was enabled by a large-scale collaborative network. Consequently, LI Y ranked first in important indicators, TC, h-index, g-index and m-index, and became a leading scholar combining both high output and high quality. WANG Y, with an absolute advantage in the number of publications, is an example of a “high-output” profile sustained by large collaborative networks.

### 3.5 Journal analysis and categories overlay

**Figure 6:**
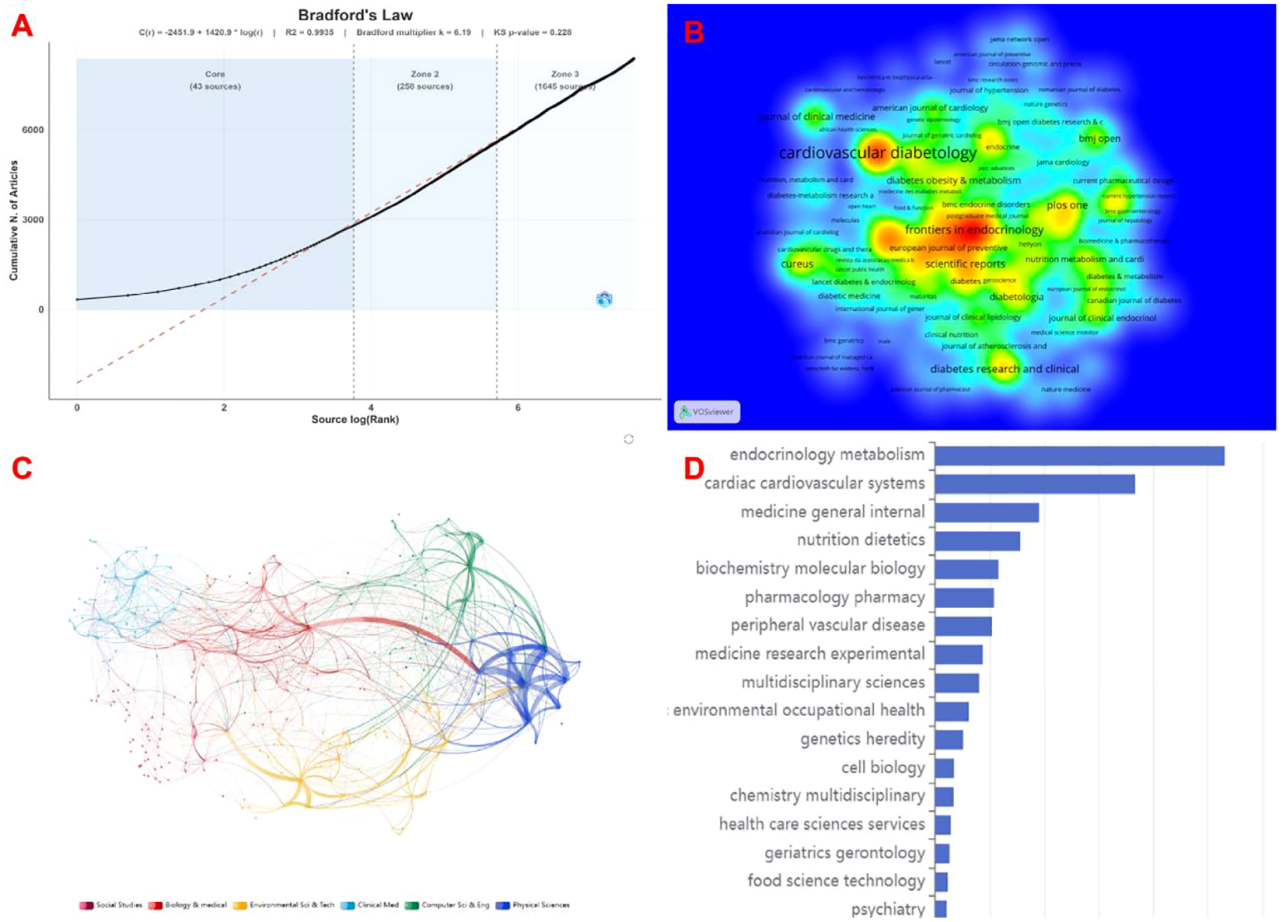
Journal distribution and disciplinary characteristics of the field. **(A)** Core journals by Bradford’s Law. **(B)** Density visualization of journals. **(C)** Categories overlay map. **(D)** Top subject categories ranked by publication count.

Bradford’s Law analysis (**Figure 6A**) revealed that all source journals could be divided into three zones; the core zone comprised 43 journals, indicating that a relatively mature core journal group has taken shape in this field.

As shown in **Figure 6B** and **Table 5**, *Cardiovascular Diabetology* led with 335 publications and also ranked first in total citations (12,123), h-index (49), and g-index (97), indicating that this journal not only served as the most prolific publication venue but also acted as the central hub of scholarly impact in the field. Notably, *Diabetes Care*, although ranking third in publication count (126), exhibited outstanding performance in impact factor (16.6) and total citations (9,082), yielding a high per-article impact and suggesting that this journal preferentially publishes high-quality landmark studies. Additionally, the *International Journal of Molecular Sciences* (79 publications, IF = 4.9) and *Nutrients* (103 publications, IF = 5.0) rankings showed that nutritional science and molecular mechanism research are becoming popular as significant publishing directions in this field.

**Table 5:** The top 10 prolific journals in the field.

Ranking	Journal	Publications	h_index	g_index	m_index	Citations	Impact factor (2025)	JCR (2025)
1	CARDIOVASCULAR DIABETOLOGY	335	49	97	4.455	12123	10.6	Q1
2	FRONTIERS IN ENDOCRINOLOGY	135	22	42	2	2121	4.6	Q1
3	DIABETES CARE	126	48	94	4.364	9082	16.6	Q1
4	DIABETES RESEARCH AND CLINICAL PRACTICE	119	23	39	2.091	2007	7.4	Q1
5	NUTRIENTS	103	41	74	3.727	5684	5.0	Q1
6	SCIENTIFIC REPORTS	93	23	37	2.091	1702	3.9	Q1
7	PLOS ONE	90	24	52	2.182	2852	2.6	Q1
8	JOURNAL OF DIABETES AND ITS COMPLICATIONS	83	22	35	2	1539	3.1	Q2
9	FRONTIERS IN CARDIOVASCULAR MEDICINE	83	18	36	2	1463	2.9	Q2
10	INTERNATIONAL JOURNAL OF MOLECULAR SCIENCES	79	30	79	2.727	7086	4.9	Q1

h-index, g-index, and m-index were calculated based on publications from each journal within the retrieved dataset. Impact Factor and JCR quartile refer to the 2025 Journal Citation Reports.

**Figure 6C** shows that, although clinical medicine formed the core, disciplines such as environmental science and computational science also intersected with the field. The field’s disciplinary profile is shown in **Figure 6D**, with Endocrinology & Metabolism dominating by a large margin, followed by Cardiac & Cardiovascular Systems and Medicine, General & Internal. Nutrition, Biochemistry & Molecular Biology, Pharmacology, and Peripheral Vascular Disease also claimed notable shares, revealing that the field has progressively extended beyond traditional clinical management to encompass molecular mechanism elucidation and nutritional interventions, thereby unfolding as a highly integrated, cross-disciplinary domain.

### 3.6 Most global cited documents

Highly cited documents serve as a critical window for identifying the knowledge base and scholarly consensus of a field. As shown in **Table 6**, the top 10 most-cited publications in our dataset accumulated 14,949 citations, forming the most influential knowledge base in this domain. These documents’ composition is instructive: out of the ten entries, six were reviews, two were clinical guidelines, one was a systematic review, and only one was an original research article. Taken together, syntheses and consensus statements accounted for 90% of the list. This distribution pattern shows that authoritative consensus documents and integrated comprehensive knowledge outcomes serve as the primary means of establishing the knowledge foundation in this field, indicating that the field has reached a mature stage marked by clinical translation and evidence integration.

**Table 6:** Top 10 highly cited publications.

Ranking	Title	Citation	Type	Journal	Year	Impact factor (2022)
1	2019 ESC Guidelines on diabetes, pre-diabetes, and cardiovascular diseases developed in collaboration with the EASD: The Task Force for diabetes, pre-diabetes, and cardiovascular diseases of the European Society of Cardiology (ESC) and the European Association for the Study of Diabetes (EASD)	3560	Guideline	*European Heart Journal*	2019	35.6
2	2019 ACC/AHA Guideline on the Primary Prevention of Cardiovascular Disease: A Report of the American College of Cardiology/American Heart Association Task Force on Clinical Practice Guidelines	2597	Guideline	*Circulation*	38.6	2019
3	Prevalence of cardiovascular disease in type 2 diabetes: a systematic literature review of scientific evidence from across the world in 2007-2017	1595	Systematic Review	*Cardiovascular Diabetology*	10.6	2018
4	Obesity Phenotypes, Diabetes, and Cardiovascular Diseases	1509	Review	*Circulation Research*	16.2	2020
5	Genetics of diabetes mellitus and diabetes complications	1379	Review	*Nature Reviews Nephrology*	39.8	2020
6	Mortality and cardiovascular disease in type 1 and type 2 diabetes	1094	Original Article	*New England Journal of Medicine*	78.5	2017
7	Clinical Update: Cardiovascular Disease in Diabetes Mellitus Atherosclerotic Cardiovascular Disease and Heart Failure in Type 2 Diabetes Mellitus - Mechanisms, Management, and Clinical Considerations	986	Review	*Circulation*	38.6	2016
8	Insulin resistance and hyperinsulinaemia in diabetic cardiomyopathy	853	Review	*Nature Reviews Endocrinology*	40	2016
9	Diabetes as a cardiovascular risk factor: An overview of global trends of macro and micro vascular complications	775	Review	*European Journal of Preventive Cardiology*	7.5	2019
10	Diabetic Cardiomyopathy	601	Review	*Circulation research*	16.2	2019

Regarding citation performance, the *2019 ESC/EASD Guidelines on diabetes, pre-diabetes, and cardiovascular diseases* ranked first with 3,560 citations, while the *2019 ACC/AHA Guideline on the Primary Prevention of Cardiovascular Disease* ranked second with 2,597 citations. These two guidelines together accounted for 6,157 citations, or 41.2% of the total number of citations received by the top 10 documents. This suggests that clinical practice guidelines serve as anchor papers in the citation network, influencing subsequent research questions, study design, and standard-setting in a paradigmatic way.

**Figure 7:**
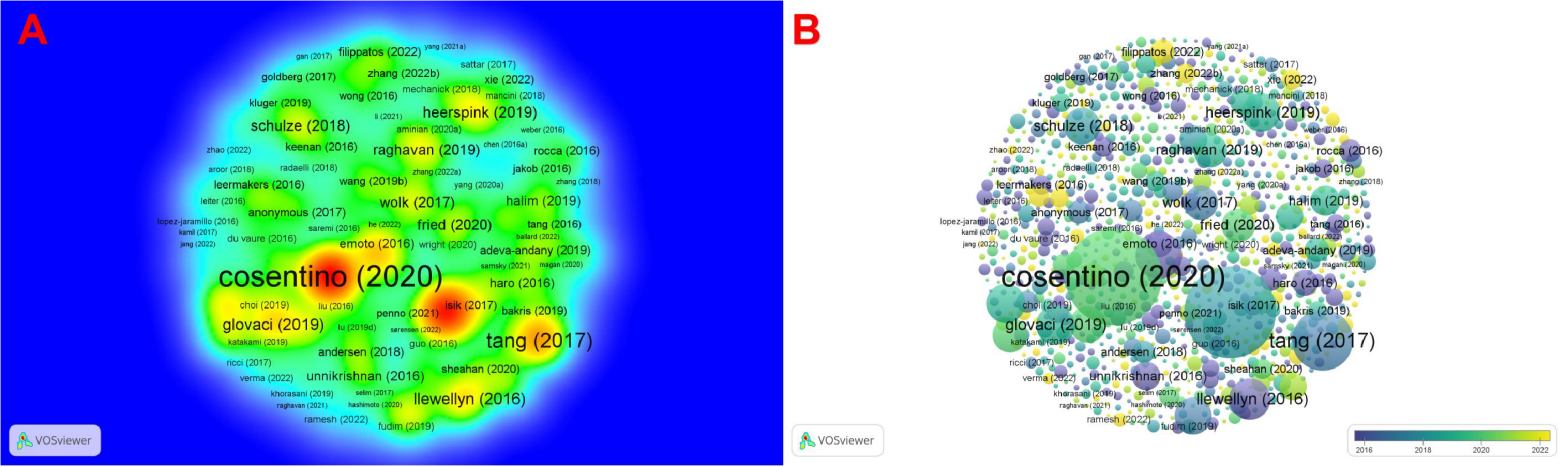
Network visualization of highly cited documents in this field. **(A)** Density visualization; **(B)** Overlay visualization.

With respect to journal distribution, the list was led by high-level cardiovascular and review journals such as Circ (2 papers), Circ Res (2 papers), Eur Heart J, Nat Rev Nephrol, Nat Rev Endocrinol and the New England Journal of Medicine.Thematically, the highly cited documents spanned three main directions: (1) clinical guidelines and prevention strategies, (2) epidemiological evidence and disease burden, and (3) pathological mechanisms and molecular basis, mirroring the field’s disciplinary trajectory of advancing from macro-level epidemiology to in-depth micro-mechanism investigations. The temporal evolution and structural locations of these documents within the knowledge network are further shown in **Figures 7A, B**. The blue-colored earlier nodes typically focus on diabetic complications and traditional cardiovascular risk factors. The more recent entries, which lean more toward yellow-green, focus more on precision-medicine techniques, updated guidelines, and new therapeutic targets. This color gradient suggests that the knowledge base of the field is transitioning from classic risk factor management toward novel intervention strategies and individualized medicine.

### 3.7 Keyword analysis and thematic evolution

Keywords condense the conceptual core of published work, and their co-occurrence patterns together with temporal trajectories illuminate the research hotspots, and emerging frontiers of a field.

#### 3.7.1 High-frequency keywords

**Figure 8:**
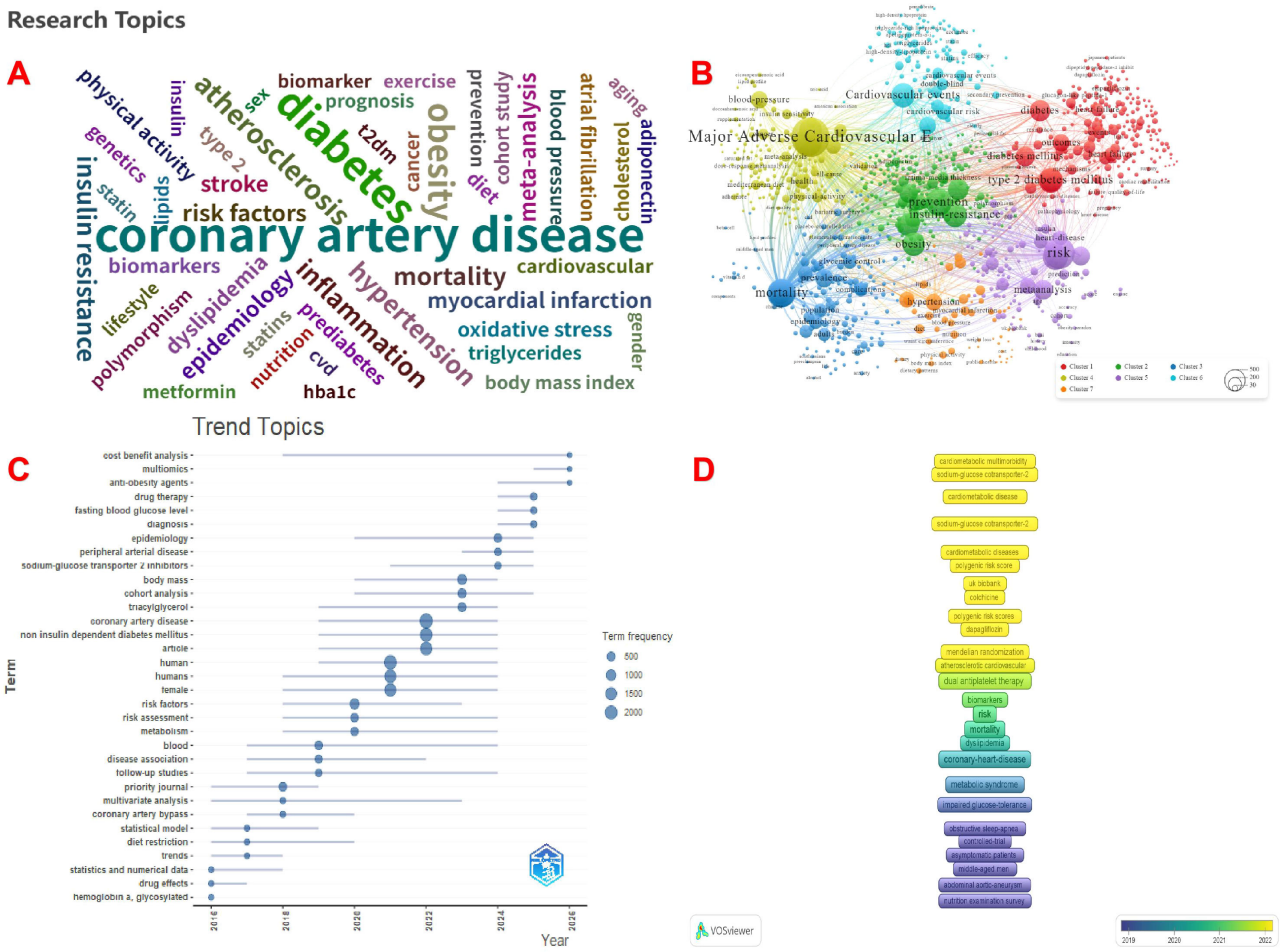
Integrative analysis of high-frequency keyword distribution, thematic clustering, and frontier evolution. **(A)** Word cloud of high-frequency keywords; **(B)** Keyword co-occurrence clustering network; **(C)** Trend topic analysis; **(D)** Timeline evolution diagram of keywords.

Word cloud analysis (**Figure 8A**) revealed that the high-frequency keywords in this field converged on four major domains: (1) clinical endpoint events (e.g., mortality, myocardial infarction); (2) risk factors (e.g., hypertension, obesity, dyslipidemia, insulin resistance); (3) pathological mechanisms and biomarkers (e.g., inflammation, atherosclerosis, oxidative stress); and (4) interventions (e.g., metformin, statins, lifestyle, nutrition, exercise, prevention).

#### 3.7.2 Keyword clustering analysis

Keyword clustering analysis (**Figure 8B**) further delineated seven relatively independent yet interrelated research communities from the high-frequency terms, each capturing a distinct dimension of scholarly exploration at the intersection of T2DM and CHD. Specifically, the red cluster centered on mechanistic studies (e.g., mechanisms, type 2 diabetes mellitus); the green cluster revolved around primary prevention (e.g., prevention, insulin resistance, obesity, lifestyle); the yellow cluster focused on cardiovascular endpoints (e.g., major adverse cardiovascular events, mortality, blood pressure, hypertension, cardiovascular events); the blue cluster concentrated on epidemiological methods and population characteristics (e.g., epidemiology, prevalence, meta-analysis); the orange cluster emphasized lifestyle modification and nutritional interventions (e.g., exercise, diet, nutrition, physical activity); the purple cluster highlighted risk prediction modeling and evidence synthesis (e.g., risk, meta-analysis, score, cohort); and the light blue cluster was devoted to secondary prevention (e.g., secondary prevention, statin, triglyceride-rich lipoproteins).

#### 3.7.3 Research frontiers and thematic evolution

Trend-topic analysis (**Figure 8C**) identified the field’s emerging hotspots. Cost-benefit analysis, multiomics, anti-obesity agents, drug therapy, and SGLT2 inhibitors showed markedly rising trajectories in recent years, signaling that precision diagnosis, multi-omic exploration, and novel glucose-lowering agents are consolidating as new research fronts.

The keyword temporal evolution map (**Figure 8D**) further delineated the chronological evolution of topics and the frontier dynamics. Viewed along the timeline, research hotspots from 2016 to 2019 centered on epidemiological investigations and risk factor management. Between 2020 and 2021, the field shifted from isolated risk-factor description toward the integrative concept of metabolic syndrome. Notably, the sustained presence of “dual antiplatelet therapy” underscored the ongoing clinical importance of antiplatelet treatment in diabetic cardiovascular comorbidity.

The uppermost tier of the timeline concentrated the most forward-looking directions, which can be distilled into five emerging streams:

1. Novel metabolic-cardiovascular agents. GLP-1 receptor agonists and SGLT2 inhibitors have evolved from pure glucose-lowering drugs into dual metabolic-cardiac interventions with proven cardiovascular benefit. Their clinical economic evaluation and real-world evidence generation are becoming active research areas (e.g., GLP-1 receptor agonist, sodium-glucose cotransporter-2, dapagliflozin, receptor agonists).
2. Precision medicine and genetic causal inference. The appearance of polygenic risk score and mendelian randomization signals a methodological pivot toward genetic risk stratification and causal inference, offering a new methodological basis for individualized prevention.
3. Artificial intelligence and big data mining: machine learning and algorithm-driven risk prediction models are emerging as methodological frontiers.
4. Cardiometabolic multimorbidity: terms such as cardiometabolic multimorbidity, cardiometabolic disease/diseases, and multimorbidity reflect a paradigm shift in research perspective from single diseases (diabetes or coronary heart disease) toward the integrated management of cardio-metabolic-multisystem comorbidities, aligning closely with the growing burden of multimorbidity in the context of global population aging.
5. Large scale cohorts and omics technologies: UK Biobank and proteomics signify that real-world big data, long-term follow-up studies, and multiomics are becoming new research priorities.

## 4 Discussion

### 4.1 Research overview

To systematically map the global research landscape of T2DM co-occurring with CHD, we used a multi-dimensional bibliometric analysis framework with the following components: (1) Annual publication trend analysis: By tracking changes in annual publication output and citations between April 2016 and April 2026, this analysis defines the developmental stage and trajectory of academic attention in the field, providing an objective reference for researchers to gauge field momentum and plan study timing; (2) Collaboration network analysis (countries/regions, institutions, and authors): This analysis identifies core research contributors and global collaboration patterns, and maps the geographic distribution of leading teams and institutions, to guide future cross-regional and cross-institutional academic partnerships; (3) Highly cited literature analysis: This analysis outlines core journal clusters in the field and pinpoints highly cited landmark publications that form the foundational knowledge base. It helps researchers quickly identify authoritative submission journals and key reference materials, improving the efficiency of literature screening; (4) Keyword co-occurrence and thematic evolution analysis: Based on keyword clustering and temporal mapping, this analysis extracts core research themes, tracks shifts in research priorities, and identifies promising emerging directions. It provides data-driven support for topic selection in future basic and clinical studies. From the temporal distribution of the knowledge map, the highly cited documents clustered conspicuously around 2019. This pattern probably reflects a major update of knowledge around 2019 when the ESC/EASD and ACC/AHA guidelines were published simultaneously, updating the consensus framework for clinical practice. At the country level, China holds a clear lead in total publication output, yet US research shows a stronger propensity for cross-border collaboration and, on average, greater citation impact. This disparity suggests that China’s share of genuinely ground-breaking original contributions and its international visibility still have potential to increase. Institutionally, the top ranks are still dominated by North American and European organizations, a reminder of the accumulated research depth of old scientific powers. Among the highly cited literature, the ESC/EASD and ACC/AHA guidelines serve as the cornerstones of the consensus framework. Careful study of international guidelines and mechanistic reviews therefore offers an effective pathway to rapidly grasp the core issues and master the academic discourse of the field.

### 4.2 Research hotspots and future trends

Building on the knowledge structure delineated above and the evolutionary trends identified in this field, we propose several underexplored and promising research directions to guide future high-value investigations and propel the field forward.

1. Comorbidity management: The prevalence of T2DM complicated by continues to climb, and a growing body of evidence demonstrates that type 2 diabetes may influence the choice of treatment for coronary heart disease (17, 18). Conventional single-disease management places significant burdens on both patients and health systems, including fragmented appointments, rising service demands, potentially contradictory lifestyle advice, and a poorer overall care experience (19–22). Accordingly, comorbidity management—streamlining care pathways, reducing silos across service departments, and thereby elevating the overall quality of health services and achieving integrated reduction of multiple risk factors—is becoming increasingly imperative (23–26). Developing clinical pathways and decision-support tools tailored to multimorbidity, and designing integrated care models, represent vital routes toward effective comorbidity management in the future (27).
2. Multi-omic technologies: The convergence of metabolomics, genomics, proteomics, transcriptomics, and microbiomics has made it feasible to decode the interplay among complex biological pathways (28–30). In this field, multi-omics approaches have already been applied to early screening and risk stratification tools, elucidation of disease mechanisms, and identification of potential therapeutic targets. Integrative multi-omic frameworks hold particular promise for tailoring prevention and treatment to individual patients, especially those with multimorbidity (31–33). However, the clinical translation of multi-omics still faces considerable hurdles: the complexity of methodologies, the challenge of integrating heterogeneous multi-omics data, and the lack of standardized protocols across laboratories and platforms (34, 35). These limitations have hampered the translation of metabolomic findings into clinical practice (36). Future work should prioritize the establishment of cost-effective, highly reproducible workflows and the validation of biomarker panels in diverse populations to support broader clinical adoption (37–39). This involves creating cross-platform, cross-population metabolomic reference intervals and creating machine learning-based multi-omics integration algorithms that combine metabolomic signatures with proteomic, clinical, and genomic variables (40, 41).

### 4.3 Molecular mechanisms of T2DM-CHD comorbidity and future perspectives

Current therapeutic strategies for T2DM-CHD comorbidity primarily target lipid metabolism or inflammatory pathways, both of which are widely considered core mechanisms of the disease. However, evidence indicates that standard lipid-lowering interventions fail to halt the progression of vascular injury specific to T2DM-CHD comorbidity. This clinical paradox suggests that the pathogenesis of T2DM-CHD comorbidity is far more complex than the traditional model of lipid deposition and chronic inflammation (42, 43). Recent frontier research has proposed a new explanatory framework for the comorbid mechanisms: oxidative stress imbalance serves as the central hub, linking mitochondrial dysfunction and cardiac energy metabolic remodeling into a vicious cycle (44–46). Oxidative stress is fundamentally a homeostatic imbalance between the generation of reactive oxygen species and reactive nitrogen species and the antioxidant defense system (47). It also constitutes the shared upstream core pathway bridging T2DM glucolipid metabolic disorders and coronary atherosclerosis. Chronic hyperglycemia aberrantly activates four major pathways: the polyol pathway, the advanced glycation end-product pathway, protein kinase C activation, and the hexosamine pathway (48). All of these pathogenic pathways depend on ROS overproduction to form positive feedback loops that continuously amplify vascular injury. Excessive ROS specifically damage the PI3K/Akt vasoprotective pathway and inhibit endothelial nitric oxide synthesis and release, serving as the initiating mechanism of coronary atherosclerosis in T2DM (49). Mitochondria are both the primary source of ROS and the primary target of oxidative stress (50). In T2DM, mitochondria sustain comprehensive structural and functional damage, manifested as mitochondrial fragmentation, cristae disruption, and decreased activity of respiratory chain complexes I, III, and IV. These changes lead to impaired mitochondrial respiration and uncoupling of oxidative phosphorylation, resulting in insufficient myocardial energy production (51–53). Simultaneously, ROS-mediated depletion of the mitochondrial thioredoxin and glutathione antioxidant systems further exacerbates oxidative injury (54). Oxidative stress-mediated multi-pathway crosstalk is the key underlying mechanism driving the development, progression, and high vulnerability of plaques in T2DM-CHD. The cardiovascular benefits of novel agents such as glucagon-like peptide-1 receptor agonists and sodium-glucose cotransporter 2 inhibitors beyond glycemic control may be attributed to their direct effects in reducing oxidative stress, improving mitochondrial function, and modulating substrate metabolism (55, 56). Looking forward, precision interventions targeting mitochondrial homeostasis hold great promise. However, safely translating mitochondrial-targeted strategies into clinical practice, and integrating multi-omics and artificial intelligence to achieve precision therapy for T2DM-CHD comorbidity, remain key bottlenecks that urgently need to be overcome in this field (57, 58).

## 5 Conclusion

In this study, we employed bibliometric analysis and systematically searched multiple databases to cover the relevant literature in this field over the past decade. Through the sustained contributions of numerous researchers, the knowledge base has undergone continuous evolution and refinement. Current research hotspots center on novel intervention strategies, artificial intelligence, and polygenic risk scores. Looking ahead, research emphasis is likely to shift toward comorbidity management and the application of multi-omics technologies, thereby advancing precision medicine and integrated care.

## Data Availability

Publicly available datasets were analyzed in this study. The datasets presented in this study can be found in online repositories. The names of the repository/repositories and search strategy can be found in the article.
